# Supplementary material for: Deep Levels and Electron Paramagnetic Resonance Parameters of Substitutional Nitrogen in Silicon from First Principles
Source: Nanomaterials (Basel). 2023 Jul 21;13(14):2123. doi: 10.3390/nano13142123 (PMC10384624; doi:10.3390/nano13142123)
Supplement: Supplementary file 1 [file nanomaterials-13-02123-s001.zip › nanomaterials-2503658-supplementary.pdf]

Supplementary materials: Deep levels and Electronic Paramagnetic Resonance parameters of substitutional nitrogen in silicon from first principles

Chloé Simha, Gabriela Herrero-Saboya, Luigi Giacomazzi, Layla Martin-Samos, Anne Hémeryck, and Nicolas Richard

I. BAND STRUCTURES OF DEFECTIVE SILICON CELLS

In the main text, the single-electron energies corresponding to defect states are taken as the Kohn-Sham (Quasi-particle) eigenvalues of the 216-atom defective cell sampled at Γ. For both the on-center and off-center configurations (Figure I), the highest occupied state corresponds to a spin up defect state, $a_1^{\uparrow}$, located within the silicon bands. The lowest unoccupied state is therefore the spin down projection of that defect state, $a_1^{\downarrow}$. As shown in the band structures of Figure I, these states do however present an artificial dispersion when k-points are considered. In the case of the on-center configuration, this dispersion is rather significant, since the highest occupied state becomes lower than the lowest unoccupied state, $\epsilon_{a_1^{\uparrow}}(\mathbf{k} = \mathrm{L}) < \epsilon_{a_1^{\downarrow}}(\mathbf{k} = \Gamma)$, resulting in a *negative band gap.* In the case of the off-center configuration, the dispersion of defect states is most notable for the $a_1^{\downarrow}$ state, and almost negligible for the $a_1^{\uparrow}$ state. This finite size effect becomes relevant when estimating fine properties of the defect, such as the g-tensor and the hyperfine structure tensor, as discussed in the main text. When evaluating deep levels, the first ionization potential shows a reasonable precision at Γ, whereas the first electronic affinity presents an energy span of ∼ 0.1 eV in the Kohn-Sham description.

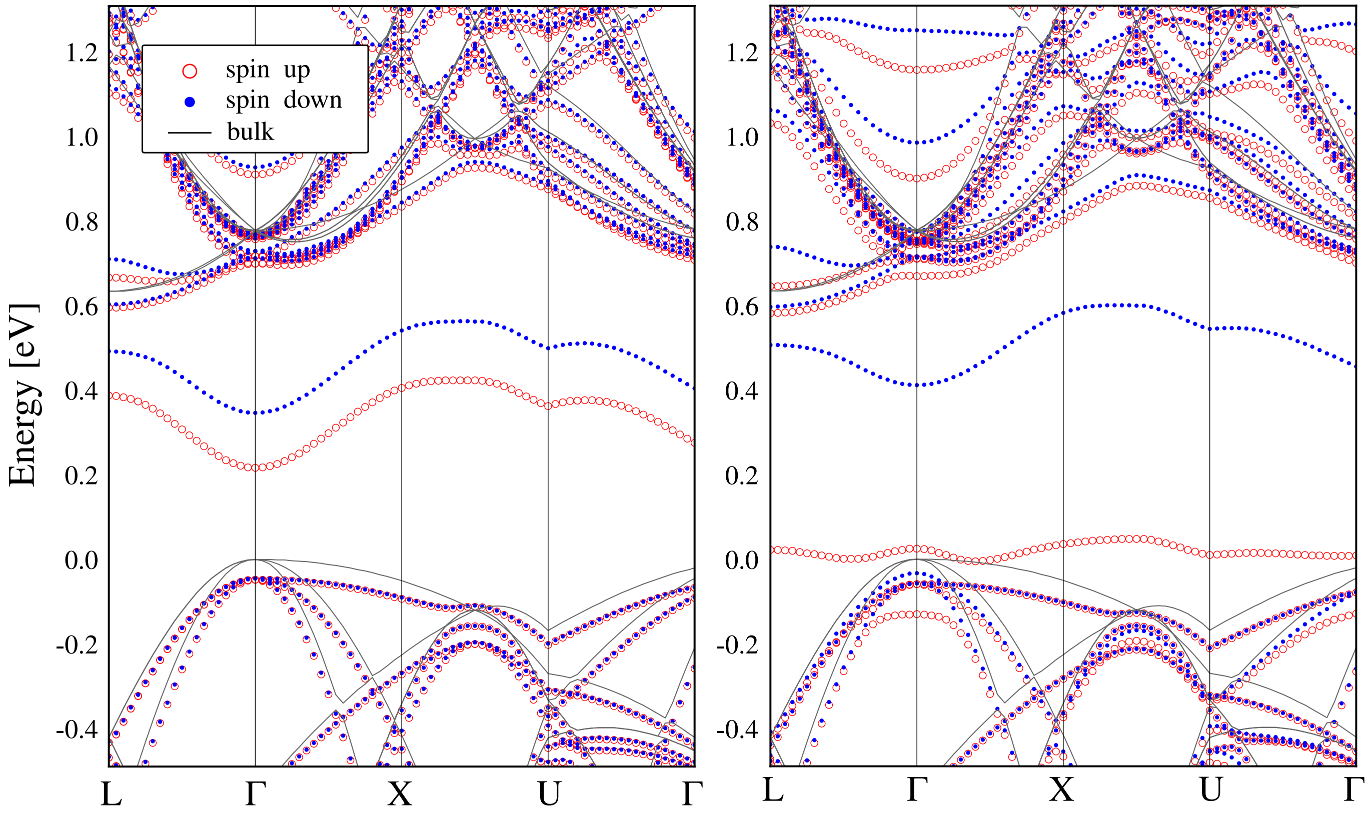

Figure S1. Band structures of substitutional nitrogen in 216-atom cells from standard PBE calculations. Kohn-Sham eigenvalues for the on-center (left) and off-center (right) configurations are shown. Spin up *versus* spin down contributions are represented in red and blue respectively. The eigenvalues corresponding to the pristine cell are depicted in black. The zero energy corresponds to highest occupied Kohn-Sham eigenvalue in the pristine cell (or the top of the valence band).
